# Orexin/hypocretin (Orx/Hcrt) transmission and drug-seeking behavior: is the paraventricular nucleus of the thalamus (PVT) part of the drug seeking circuitry?

**DOI:** 10.3389/fnbeh.2012.00075

**Published:** 2012-11-09

**Authors:** Rémi Martin-Fardon, Benjamin Boutrel

**Affiliations:** ^1^Molecular and Integrative Neurosciences Department, The Scripps Research InstituteLa Jolla, CA, USA; ^2^Department of Psychiatry, Center for Psychiatric Neuroscience, Lausanne University HospitalLausanne, Switzerland

**Keywords:** paraventricular nucleus of the thalamus, orexin, hypocretin, drug-seeking behavior, natural reward

## Abstract

The orexin/hypocretin (Orx/Hcrt) system has long been considered to regulate a wide range of physiological processes, including feeding, energy metabolism, and arousal. More recently, concordant observations have demonstrated an important role for these peptides in the reinforcing properties of most drugs of abuse. Orx/Hcrt neurons arise in the lateral hypothalamus (LH) and project to all brain structures implicated in the regulation of arousal, stress, and reward. Although Orx/Hcrt neurons have been shown to massively project to the paraventricular nucleus of the thalamus (PVT), only recent evidence suggested that the PVT may be a key relay of Orx/Hcrt-coded reward-related communication between the LH and both the ventral and dorsal striatum. While this thalamic region was not thought to be part of the “drug addiction circuitry,” an increasing amount of evidence demonstrated that the PVT—particularly PVT Orx/Hcrt transmission—was implicated in the modulation of reward function in general and several aspects of drug-directed behaviors in particular. The present review discusses recent findings that suggest that maladaptive recruitment of PVT Orx/Hcrt signaling by drugs of abuse may promote persistent compulsive drug-seeking behavior following a period of protracted abstinence and as such may represent a relevant target for understanding the long-term vulnerability to drug relapse after withdrawal.

## Introduction

Drug addiction is a chronically relapsing disorder characterized by compulsive drug seeking and use (O'Brien and McLellan, 1996; Leshner, 1997; O'Brien et al., 1998; McLellan et al., 2000). Advances have been made in elucidating the neurocircuitry that mediates craving and drug seeking. Functional brain imaging in humans (e.g., Miller and Goldsmith, 2001; Goldstein and Volkow, 2002; Daglish and Nutt, 2003) and brain site-specific manipulations in animals (e.g., Everitt et al., 2001; Cardinal et al., 2002; See, 2002; Weiss, 2005) implicate interconnected cortical and limbic brain regions in response to drug cue-, drug priming-, and stress-induced reinstatement. Major components of this circuitry include the medial prefrontal cortex (mPFC), basolateral amygdala (BLA), central nucleus of the amygdala (CeA), bed nucleus of the stria terminalis (BNST), hippocampus, nucleus accumbens (NAC), and, more recently, dorsal striatum, which is thought to participate in consolidating stimulus-response habits via the engagement of corticostriatal loops (Everitt et al., 2001; McFarland and Kalivas, 2001; Ito et al., 2002; Kalivas and Volkow, 2005; Belin and Everitt, 2008; Steketee and Kalivas, 2011). However, unclear is what differentiates neural signaling related to normal appetitive behavior *vs*. compulsive behavior that results from long-term drug exposure. There is an overlap between the brain regions implicated in the processing of natural rewards and drugs of abuse, and it is thought that neural circuitry encoded for natural rewards is usurped by drugs of abuse. Neuroplasticity within this neural circuitry is believed to be responsible for the maladaptive (compulsive) behavior characteristic of addiction (Kelley and Berridge, 2002; Aston-Jones and Harris, 2004; Kalivas and O'Brien, 2008; Wanat et al., 2009), which may account for the interindividual vulnerability to drug abuse.

## Recruitment of the Orx/Hcrt system by drugs of abuse

About 10 years ago, the orexin/hypocretin (Orx/Hcrt) system, already known to regulate a wide range of physiological processes, was shown to be recruited by drugs of abuse. Indeed, orexin A (Orx-A or hypocretin-1 [Hcrt-1]) and orexin B (Orx-B or hypocretin-2 [Hcrt-2]) were initially considered hypothalamic neuropeptides that regulate feeding, energy metabolism (Sakurai et al., 1998; Edwards et al., 1999; Haynes et al., 2000, 2002; Willie et al., 2001; Teske et al., 2010), and arousal (Sutcliffe and de Lecea, 2002; Taheri et al., 2002). Among the two Orx/Hcrt receptors identified, Hcrt-r1 binds to Orx-A/Hcrt-1 with 20–30 nM affinity but has much lower affinity (10–1000-fold lower) for Orx-B/Hcrt-2, and Hcrt-r2 binds to both peptides with similar affinity (in the 40 nM range; Sakurai et al., 1998; Ammoun et al., 2003; Scammell and Winrow, 2011). Orx/Hcrt cell bodies are essentially found in the lateral hypothalamus (LH), a brain region long associated with reward and motivation (for review, see DiLeone et al., 2003), perifornical hypothalamus (PFA), and dorsomedial hypothalamus (DMH). Hypothalamic Orx/Hcrt neurons project to brainstem nuclei where they are considered to play a major role in the regulation of arousal and modulation of stress responses (Baldo et al., 2003; Winsky-Sommerer et al., 2004). They also project to the paraventricular nucleus of the thalamus (PVT), NAC shell (NACsh), ventral pallidum (VP), ventral tegmental area (VTA), CeA, and BNST (Peyron et al., 1998; Baldo et al., 2003). A past conjecture suggested a dichotomy in Orx/Hcrt function, with Orx/Hcrt neurons in the LH regulating reward processes and Orx/Hcrt neurons in the PFA and DMH being mostly involved in the regulation of arousal and stress responses (Harris and Aston-Jones, 2006). However, recent evidence opposes this interpretation because similar patterns of Fos-positive Orx/Hcrt cells were observed in both the PFA/LH and DMH in rats exposed to contextual stimuli previously paired with ethanol availability (Dayas et al., 2008). Furthermore, medial and lateral Orx/Hcrt cells were shown to project to both the locus coeruleus and VTA, confirming that convergent projections from different Orx/Hcrt populations to these two brain areas may strengthen the temporal association between stress, arousal, and reward-seeking, thus optimizing goal-oriented behavioral strategies (Calipari and Espana, 2012; Gonzalez et al., 2012).

In addition to their involvement in the regulation of natural rewards, recent evidence showed that hypothalamic Orx/Hcrt neurons played a significant role in the modulation of reward function and, particularly, drug-directed behaviors (Harris et al., 2005). Hypothalamic Orx/Hcrt neurons become activated by stimuli associated with food, morphine, cocaine, and ethanol (Harris et al., 2005; Dayas et al., 2008; Martin-Fardon et al., 2010; Jupp et al., 2011). Similarly, the expression of conditioned place preference induced not only by food but also by morphine and cocaine is associated with activation of Orx/Hcrt neurons in the LH (Harris et al., 2005) likely due to the stimulation of LH Orx/Hcrt by rostral lateral septum afferents (Sartor and Aston-Jones, 2012). Consistent with these observations, intra-VTA microinjection of Orx-A produces a renewal of morphine-induced conditioned place preference, whereas administration of the Hcrt-r1 antagonist *N*-(2-methyl-6-benzoxazolyl)-*N*′-1,5-n-aphthyridin-4-yl urea (SB334867) decreases the expression of morphine-induced conditioned place preference (Harris et al., 2005). SB334867 also blocks the acquisition of cocaine-induced behavioral sensitization and potentiation of excitatory currents induced by cocaine in VTA dopamine neurons (Borgland et al., 2006). Intra-VTA administration of SB334867 reduces the motivation to self-administer cocaine and attenuates the cocaine-induced enhancement of dopamine signaling in the NAC (Espana et al., 2010). Blockade of Hcrt-r1 decreases ethanol (Lawrence et al., 2006) and nicotine (Hollander et al., 2008) self-administration, inhibits cue-induced reinstatement of ethanol (Lawrence et al., 2006) and cocaine (Smith et al., 2010) seeking, and attenuates stress-induced reinstatement of cocaine and ethanol seeking (Boutrel et al., 2005; Richards et al., 2008).

Thus, behavioral and functional evidence indicates a role for Orx/Hcrt signaling in the motivational effects of cocaine and other drugs of abuse (Borgland et al., 2006; Bonci and Borgland, 2009; Thompson and Borgland, 2011), but questions remain about what differentiates Orx/Hcrt signaling related to normal appetitive behavior *vs*. compulsive behavior that results from long-term drug exposure.

## Differential recruitment of the Orx/Hcrt system by drugs of abuse and natural rewards

One hypothesis concerning the control of drug-seeking behavior is that the neural circuits that mediate these effects are common motivational circuits that are more robustly activated by drug-related stimuli and not specific to addiction-related events. This activation that normally governs responding for natural rewards creates new motivational states or tilts processes that normally govern responding for natural rewards toward drug-directed behavior (Kelley and Berridge, 2002). Considered to orchestrate the appropriate levels of alertness required for the elaboration and execution of goal-oriented behaviors, Orx/Hcrt is one legitimate candidate for further investigating how a system normally involved in the regulation of motivation and arousal may trigger a pathological state that elicits compulsive craving and relapse to drug seeking (Boutrel et al., 2010).

Evidence has accumulated that the Orx/Hcrt system is, in fact, more strongly engaged by drugs of abuse compared with natural non-drug reinforcers. For example, it has been shown that SB-334867 treatment significantly reduced responding for ethanol but not sucrose under a progressive-ratio schedule of reinforcement (Jupp et al., 2011). An even more striking observation was that, although the stimuli conditioned to cocaine, ethanol, and a conventional reinforcer were shown to equally elicit reinstatement, SB334867 treatment selectively reversed conditioned reinstatement induced by a cocaine- or ethanol-related stimulus but had no effects on the same stimulus conditioned to a conventional reinforcer (sweetened condensed milk [SCM] or SuperSac [3%/0.125%, w/v, glucose/saccharin]; Martin-Fardon and Weiss, 2009, 2012; Martin-Fardon et al., 2010).

The pharmacological and neural mapping data are difficult to reconcile with the role of the Orx/Hcrt system in behavior motivated by food (i.e., a natural reward) and its more recently discovered role in drug reward. One hypothesis concerning the control of drug-seeking behavior is that the neural circuits that mediate the effects of drug cues are not specific to addiction-related events but rather are common motivational circuits that are more robustly activated by drug-related stimuli. This activation will create new motivational states or tilt processes that normally govern responding for natural rewards toward drug-directed behavior (Kelley and Berridge, 2002). Drugs of abuse may produce this effect by neuroadaptively altering the neural systems that regulate motivation directed toward natural rewards. Evidence of drug-induced dysregulation of the Orx/Hcrt system exists for alcohol. For example, prepro-orexin mRNA is up-regulated in the LH in inbred alcohol-preferring (iP) rats following chronic ethanol consumption (Lawrence et al., 2006). A possibility derived from this hypothesis is that the Orx/Hcrt system may, over the course of repeated drug use, acquire a preferential role in mediating the effects of stimuli conditioned to drugs of abuse *vs*. natural rewards. Consequently, one explanation for the preferential role of SB334867 in conditioned reinstatement for drugs *vs*. non-drugs could be that drugs neuroadaptively alter the neural systems that regulate motivation normally directed toward natural rewards that is revealed by pharmacological (e.g., SB334867) manipulations.

Maladaptive recruitment of the Orx/Hcrt system by drugs of abuse is also suggested by findings that described neuroadaptive changes within the VTA. For example, voluntary cocaine and natural reward self-administration induces a common, short-lasting neuroadaptation in VTA dopaminergic neurons (i.e., increased glutamatergic function; Chen et al., 2008). However, this enhanced synaptic strength persists and is resistant to extinction in rats that self-administer cocaine only and not in rats that self-administer a non-drug reinforcer (Chen et al., 2008). Interestingly, several lines of evidence suggest that the participation of the VTA in cocaine-induced neuronal and behavioral changes requires Orx/Hcrt inputs. For example, activation of Hcrt-r1 in the VTA is necessary for the development of cocaine-induced locomotor sensitization (Borgland et al., 2006), and Orx-A/Hcrt-1-mediated *N*-methyl-D-aspartate (NMDA) receptor plasticity in the VTA is increased in rats that self-administer cocaine (Borgland et al., 2009). Additionally, short-lasting neuroadaptations in VTA dopaminergic neurons induced by high-fat chocolate food pellets have been described (Borgland et al., 2009), suggesting that the Orx/Hcrt-VTA system initially participates in the regulation of the motivation to obtain potent reinforcers in general (i.e., drug or highly palatable food). In contrast, drug-induced neuroadaptation of the Orx/Hcrt-VTA system is long-lasting, an effect that may be linked to the possible tilting of this system toward promoting and controlling drug-directed behavior.

## Implication of Orx/Hcrt-PVT transmission in maladaptive (drug-seeking) behavior

Anatomically, it has been shown that the PVT is the target of numerous hypothalamic peptides involved in energy homeostasis (Freedman and Cassell, 1994; Otake, 2005), including Orx/Hcrt (Kirouac et al., 2005, 2006; Ishibashi et al., 2005). It is believed that the PVT plays a key role in energy homeostasis, arousal, temperature modulation, endocrine regulation, and reward (Bhatnagar and Dallman, 1998, 1999; Van Der Werf et al., 2002; Kelley et al., 2005; Parsons et al., 2006). Specifically and particularly important for this review, lesions of the PVT were shown to increase feeding behavior and body weight (Bhatnagar and Dallman, 1999) while attenuating the increases in both locomotor activity and blood corticosterone levels normally seen during the anticipation of food reward (Nakahara et al., 2004). Furthermore, the PVT was shown to be critically involved in mediating the effects of Orx/Hcrt on brain dopamine levels and reward-based feeding behaviors (Choi et al., 2012). These findings strongly suggest an important role for Orx/Hcrt-PVT signaling in food intake regulation.

**Figure 1:**
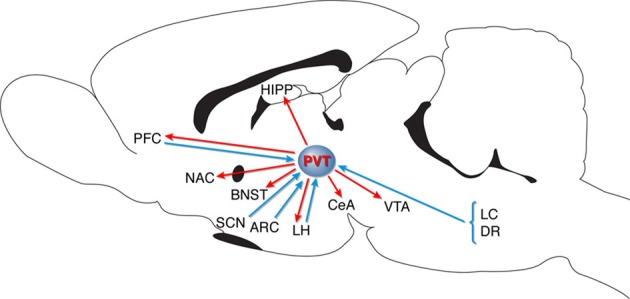
**Schematic diagram representing PVT connectivity.** PVT, paraventricular nucleus of the thalamus; LC, locus coeruleus; DR, dorsal raphe; VTA, ventral tegmental area; CeA, central nucleus of the amygdala; LH, lateral hypothalamus; ARC, arcuate nucleus; SCN, suprachiasmatic nucleus; BNST, bed nucleus of the stria terminalis; NAC, nucleus accumbens; PFC, prefrontal cortex; HIPP, hippocampus (for details, see Kelley and Berridge, 2002; Kelley et al., 2005; Kirouac et al., 2005; Parsons et al., 2006; Hsu and Price, 2009; Martin-Fardon et al., 2010; Li and Kirouac, 2012).

A major Orx/Hcrt projection exists from the LH/PFA to the PVT (Kirouac et al., 2005; Parsons et al., 2006), and the PVT has been proposed to be a key relay (see Figure 1), gating Orx/Hcrt-coded reward-related communication between the LH/PFA and both the ventral and dorsal striatum (Kelley et al., 2005). This “hypothalamic-thalamic-striatal axis” may have evolved to prolong central motivational states and promote feeding beyond the fulfillment of immediate energy needs, thereby creating energy reserves for potential future food shortages (Kelley et al., 2005). With regard to “drug seeking-related brain regions,” it is important to note that the PVT specifically projects to the CeA, BNST, NAC, VTA, and hippocampus (e.g., Kelley and Berridge, 2002; Kelley et al., 2005; Parsons et al., 2006; Hsu and Price, 2009; Martin-Fardon et al., 2010). Finally, recent data have shown that the PVT receives projections from the PFC, suggesting that these connections could modulate the expression of emotional behaviors (Li and Kirouac, 2012).

Although not usually thought of as part of drug-seeking neurocircuitry, direct and indirect findings recently supported a role for Orx/Hcrt-PVT signaling in drug-oriented behaviors. First, acute nicotine treatment was shown to activate Orx/Hcrt neurons that project to the basal forebrain and PVT, supporting a role for the Orx/Hcrt system in mediating certain aspects of nicotine-elicited wakefulness rather than proving a role for Orx/Hcrt-PVT signaling in tobacco addiction (Pasumarthi and Fadel, 2008), but such a link cannot be underestimated until proven false. Second, Orx/Hcrt peptides within the PVT have been suggested to regulate negative emotional states (Li et al., 2010a,b). Orx/Hcrt-PVT signaling was also shown to be critically involved in the expression of conditioned place aversion to morphine withdrawal (Li et al., 2011). These latter two observations may support a role for Orx-Hcrt signaling within the PVT in the negative emotional state that is putatively responsible for triggering the urge to seek drug during withdrawal of after a period of abstinence. A more direct observation confirmed that drug-related contextual cues activate Orx/Hcrt neurons (Dayas et al., 2008). Indeed, significantly larger numbers of Fos-positive hypothalamic Orx/Hcrt neurons were seen in rats exposed to contextual stimuli previously associated with ethanol availability compared with rats exposed to the same stimulus previously paired with non-reward. Moreover, presentation of the ethanol-related stimuli also increased the number of Fos-positive PVT neurons, and these neurons were closely associated with Orx/Hcrt fibers (for additional details, see Dayas et al., 2008). Other evidence supports a role for Orx/Hcrt projections from the LH to PVT in regulating drug-seeking behavior. Recent data confirmed that context-induced reinstatement of alcoholic beer seeking is associated with recruitment of a PVT-ventral striatum pathway and that excitotoxic lesion of this structure (Hamlin et al., 2009) or discrete administration of a κ opioid receptor agonist (Marchant et al., 2010) prevents context-induced reinstatement of alcohol seeking. In conclusion, it is hypothesized that maladaptive recruitment of the Orx/Hcrt system by drugs of abuse may tilt its function toward excessive drug-directed behavior, which may explain the increased sensitivity of this peptidergic system to antagonist interference with drug-seeking behavior as opposed to behavior directed toward natural rewards.

Furthermore, it was recently shown that although Orx/Hcrt microinjections into the PVT exerted a priming-like effect, reinstating both extinguished cocaine- and SCM-seeking behavior, Orx/Hcrt produced (1) two different dose-response functions for cocaine seeking *vs*. SCM seeking and (2) a stronger reinstatement of cocaine seeking *vs*. SCM seeking at moderate doses (Martin-Fardon et al., 2011). This observation suggests of a leftward shift of the Orx-A/Hcrt-1 dose-response curve in cocaine-experienced animals, implying that cocaine produced a dysregulation of Orx/Hcrt-PVT transmission that is revealed following exogenous Orx/Hcrt administration. Moreover, recent findings have demonstrated that discrete inactivation of the PVT with tetrodotoxin (TTX) prevented cocaine priming-induced reinstatement (James et al., 2010), further implicating this thalamic structure in drug-seeking behavior, although the same researchers claimed that Orx/Hcrt-1 receptor signaling within the VTA but not PVT was critical in the regulation of cue-induced reinstatement of cocaine-seeking behaviors (James et al., 2011).

## Conclusion/perspectives

Currently, the available therapeutic approaches fail to completely treat and address the compulsive nature of drug seeking and drug taking associated with addiction. Evidence indicates that dysfunctional Orx/Hcrt transmission contributes to drug seeking *vs*. natural reward seeking, and an increasing amount of data has now identified the PVT, a brain region not usually included in the neurocircuitry of addiction, to be recruited by drugs of abuse, opening up a new area of targets for efficient pharmacotherapy.

Notably, however, drug addiction is often associated with increased drug consumption that can modify the pharmacological profile of promising therapeutic agents, possibly resulting in drug-induced neuroadaptation (for review, see Kalivas and O'Brien, 2008; Moussawi et al., 2009). For instance, following extended-access cocaine self-administration (6 h/day), it was shown that (-)-2-oxa-4-aminobicylco[3.1.0]hexane-4,6-dicarboxylic acid (LY379268), a metabotropic glutamate receptor (mGluR) 2/3 agonist, became more efficient at preventing anxiety-like behavior and decreasing cocaine self-administration (Aujla et al., 2008; Hao et al., 2010), whereas the effects of an mGluR5 antagonist, 3-[(2-methyl-1,3-thiazol-4-yl)ethynyl]piperidine (MTEP), were blunted (Hao et al., 2010). A similar behavioral pharmacological profile was observed in animals that had a history of alcohol dependence, in which LY379268 and MTEP dose-dependently reduced both alcohol self-administration and the reinstatement of alcohol seeking induced by footshock stress, but LY379268 was more effective than MTEP in inhibiting both behaviors in postdependent animals compared with non-dependent animals (Sidhpura et al., 2010). Consequently, considering the importance of relapse prevention in postdependent individuals, important issues that require further research are to identify (1) whether Orx/Hcrt-PVT transmission becomes further dysfunctional following dependence and (2) the most effective pharmacological tools (i.e., Hcrt-r antagonists) for relapse and craving prevention in postdependent individuals.

### Conflict of interest statement

The authors declare that the research was conducted in the absence of any commercial or financial relationships that could be construed as a potential conflict of interest.
